# Teenage Body Image Perception, Body-shaping Behavior, and Body Composition With Respect to Use of “Fitspiration”: Exploratory Investigation Study

**DOI:** 10.2196/70964

**Published:** 2025-11-06

**Authors:** Elisabeth Höld, Theres Rathmanner, Mario Heller

**Affiliations:** 1 Institute of Health Sciences, Department of Health, St. Pölten University of Applied Sciences, Campus-Platz 1, St. Pölten, 3100, Austria, 43 2742 313 228 ext 572; 2 Institute of Creative\Media/Technologies, Department of Media and Digital Technologies, St. Pölten University of Applied Sciences, St. Pölten, Austria

**Keywords:** social media, #fitspiration, exploratory investigation study, adolescents, body image perception, body-shaping behavior, dieting, working out

## Abstract

**Background:**

The social media trend #fitspiration aims to positively impact its users’ health, but studies have shown detrimental effects, as it mainly involves stereotypical and barely achievable body images and health behaviors. During puberty, adolescents form their identity and body image, making it essential to examine social media’s influence on their health.

**Objective:**

The exploratory investigation study, part of the mixed methods study in the project FIVE (#Fitspiration Image Verification), sought initial insights into how #fitspiration consumption may affect adolescents’ body image perception, dieting, and exercise behaviors.

**Methods:**

In total, 86 adolescents (N=310, age range 14-18 years; n=42 females) attending upper secondary schools in Eastern Austria took part in an online questionnaire concerning the use of #fitspiration, body image perception, and body-shaping behaviors (dieting and working out) and bioelectrical impedance measurements to assess body composition. Participants have been classified as #fitspiration users (n=27), nonusers (n=51), and former users (n=8). We compared body image perceptions and body-shaping behaviors of #fitspiration user groups sex-specifically with the fat mass index (FMI) and the fat-free mass index (FFMI).

**Results:**

The results indicated that the amount of muscularity was of greater importance for all participants than thinness because, and even though the majority were of normal weight (76/86, 88%) and had an average to high FFMI (62/86, 92%), only 6% (5/86) of all participants thought that their amount of muscle mass was appropriate, while 43% (37/86) of all participants rated their body weight as okay. However, this outcome seemed to be of greater significance for #fitspiration users, especially males. While male participants seemed to be more dissatisfied with their amount of muscularity and worked out more often (female: 24/42, 57%, male: 35/44, 80%), female participants seemed to be more dissatisfied with their body weight and were dieting (female: 10/42, 24%; male: 6/44, 14%). Generally, none of the #fitspiration users (0/27, 0%) answered that her or his amount of muscle mass was okay, although all of them had an average or high FFMI (27/27, 100%). Participants assessed their body weight and body fat more precisely (54/86, 63% matched FMI) than their muscle mass (27/86, 31% matched FFMI).

**Conclusions:**

Our findings regarding the body ideals of adolescents are in line with the results of large-scale cross-sectional studies, indicating that they aspire to achieve a toned (and, for females, thin) body. The fact that none of the #fitspiration users were satisfied with their level of muscle mass raises the question of whether #fitspiration may perpetuate this ideal. The line between eating disorders and body dysmorphia might be very thin, especially during puberty. Therefore, our findings can be used to raise awareness of the speculated impact of #fitspiration on adolescents as a source for body ideals and consequently for body image perception and body-shaping behavior.

## Introduction

### Adolescent Body Image Perception

Body image is a multidimensional construct based on perceptions and attitudes toward one’s body and physical appearance [1]. Body image perception *“*includes body size assessment (how a person perceives his or her body), body attractiveness estimation (what is the type of body that a person considers most attractive), and perceptions related to one’s own body shape and size” [2,3]. In Western countries, the focus is on body shape and weight and the individual’s perceived personal responsibility for it [4].

During puberty, adolescents must adjust to significant bodily changes. By contrast to cognitive development, physical changes, such as the growth of a beard or breasts, are visible to everyone, including family, peer groups, and strangers if pictures are posted on social media.

Therefore, these physical changes are accompanied by a broad range of emotions and shaped by social and sociocultural influences [5]. Consequently, adolescents are a vulnerable group, and there is growing evidence that body image, body satisfaction, and body dissatisfaction affect their physical and psychosocial health, leading to, for example, unhealthy eating behaviors, lack of physical activity [6], eating disorders, or depressive symptoms [7,8].

### Impact of Visually Oriented Social Media on Body Image Perception

Photos and videos are of great significance on social media, as they fulfill the function of self-representation. Appearance is crucial for young people, and their body is part of this self-presentation. It is an expression of lifestyle and serves as a means of differentiation and belonging to groups [9]. Posed and edited social media pictures provide the unique possibility for appearance-based social comparison [10]. Consequently, both—the active use like posting selfies and passive use, like viewing pictures of others on social media—show clear associations with body dissatisfaction [7,11,12], depressive and disordered eating symptoms, and anxiety [7,8].

During the past years, visually oriented social media like Instagram (Meta Platforms, Inc) have become more popular and seem to have a greater impact on appearance comparisons, body image and consequently body image perception, body dissatisfaction [13-15], and eating disorders [16] than text-centered ones like Facebook (Meta Platforms, Inc [17,18]).

### #Fitspiration

The social media trend #fitspiration [19] mainly relates to an athletic appearance and how it can be achieved through diet and exercise [20]. Although #fitspiration aims to have a positive impact on health, it is also associated with detrimental effects, as it mainly involves stereotypical and barely achievable bodily standards [21].

Therefore, it has a high potential for physical appearance comparison. A recent review shows that #fitspiration images have a negative effect on body image, which can be linked, firstly, to the demand for self-control implied in #fitspiration images and, secondly, to the discomfort experienced by individuals when they try to achieve these unrealistic body images through hard training and restricted dietary behavior [22]. An Italian quasi-experimental intensive longitudinal study examined the impact of daily exposure to #fitspiration Instagram content on young women’s mood and body image and was associated with the highest rates of growth of negative mood and appearance comparison [23]. Furthermore, #fitspiration is also associated with a greater internalization of the muscular ideal, which further supports body dissatisfaction. Consequently, #fitspiration can lead to excessive exercising and disordered eating [22]. On the other hand, an Austrian-controlled longitudinal experiment with young adult women observed a positive impact of #fitspiration content on weight satisfaction and healthy eating behavior over 22 days compared to content from a nonbody-focused influencer [24].

Most of the literature addresses the influence of #fitspiration on young adults. Furthermore, mainly women, especially educated women, were study participants [22]. To date, there seems to be limited research on the impact of this trend on adolescents, which is surprising given that the teenage years are highly relevant for the development of body image perception and health behavior. This makes adolescents highly vulnerable to potential negative influences. Besides, most of the published study results describe changes in body image or body dissatisfaction in relation to #fitspiration use only [22,25-27].

To our knowledge, there is no study in which the gender-specific influence of #fitspiration on the body image perception of adolescents has been linked to a set of measured body composition parameters. Consequently, the purpose of this study is to describe participants’ subjective assessment of body image perception and body-shaping behavior together with objective measurements of body composition parameters in adolescents. Therefore, we can objectively rate the self-assessment of teenage body image perception against the background of #fitspiration use and gender.

## Methods

### The project #Fitspiration Image Verification

This study was part of the interdisciplinary project FIVE (#Fitspiration Image Verification [28]), which was conducted by St. Pölten University of Applied Sciences in Austria. Spanning from 2021 to 2024, it involved the Institute of Health Sciences and the Institute of Creative Media/Technologies, an external gender expert (Bettina Prokop), and an educational publisher (Hölzel Verlag GmbH). FIVE aimed to gain first insights into the effects of #fitspiration on adolescents attending upper secondary schools and developed a blended-learning course for upper secondary schools with an image forensics tool as the core element. The course aims to empower pupils to navigate critically and competently through social media. A detailed description of the development of the blended-learning course is published elsewhere [29].

As a first step of the project FIVE, an in-depth exploratory mixed methods study was conducted aiming to analyze the social media phenomenon #fitspiration and how it might affect adolescents. This mixed methods study consisted of 3 parts. The first part was a #fitspiration content analysis on Instagram, the second part was an online questionnaire, and the third part consisted of on-site anthropometric and bioelectrical impedance analysis (BIA) measurements, focus group discussions, as well as eye-tracking and biosignal measurements. This paper focuses on the body image perception-related part of the online questionnaire (part 2) and the corresponding parts of the on-site anthropometric and BIA measurements (part 3). Data collection took place from March to September 2022 at participating schools during regular teaching time. The target population consisted of pupils from upper secondary schools in Eastern Austria (Lower Austria and Vienna). The sampling frame was the network of the St. Pölten University of Applied Sciences, where schools expressed their interest in collaborating on this research project.

### Online Questionnaire

The online questionnaire had 3 main topics next to sociodemographic information. These topics were social media and #fitspiration use, body image perception, and body-shaping behavior, which are all explained in detail below.

#### Sociodemographic Information

We asked participants about their age, gender, living situation, and living area. We used the Family Affluence Scale (FAS) [30,31] to describe the socioeconomic status. Based on the United Nations Economic Commission for Europe (UNECE) recommendations [32], we defined migration background as both parents having been born outside of Austria.

#### Social Media and #Fitspiration Use

We asked the participants to rate the frequency of their social media use, both generally and with regard to #fitspiration specifically, the latter one based on the categories of [33] (1=“I have never thought about following such content or influencers”; 2=“I have thought about following such content or influencers. But I haven’t done it yet”; 3=“I thought about following such content or influencers. But I decided against it”; 4=“I follow such content or influencers and will continue to do so”; 5=“I used to follow such content or influencers, but I stopped”).

### Body Image Perception

Based on the “Health Behaviour in School-aged Children (HBSC)” study [34], we asked participants how they perceived their own body weight on a 5-point scale ranging from 1 (“much too thin”) to 5 (“much too fat”). Based on this item characteristic, we asked participants to likewise rate their muscle mass on a 5-point scale ranging from 1 (“far too unmuscular”) to 5 (“far too muscular”).

### Body-Shaping Behavior

Based on the HBSC study [34], we asked participants whether they were currently on a diet or did something else to lose weight (“Yes”; “No, I need to put on weight”; “No, my weight is fine”; “No, but I should lose some weight”). Based on this 4-item characteristic, we asked participants whether they were currently working out or doing something else to build muscle (“Yes”; “No, but I should build muscle”; “No, my amount of muscle mass is okay”; “No, because I should reduce muscle”).

The questionnaire consisted of 38 questions and could be answered in about 20-30 minutes. The usability, technical functionality, and completion time of the electronic questionnaire were tested by 3 students from the bachelor program in dietetics, and the revised final version was carried out online using LimeSurvey (version 3.27; LimeSurvey GmbH). Therefore, the project manager created individualized links for each participant. These links were sent to the participants via email by the project manager or were distributed by the school app or manually in class by the teacher. The questionnaires were available for 3 months and were answered during school time. LimeSurvey data export was used to store raw data as an SPSS (IBM Corp) file for further analysis.

### Anthropometric and BIA Measurements

The anthropometric and BIA measurements determined with the seca mBCA 555 (seca GmbH & Co. KG) took place in the morning in schools, in a fasting state, and with light clothing. The device measures body weight with a scale, body height with ultrasound length measurement, and body composition using BIA in 1 step. BIA analysis is an electrical resistance measurement on the human body, which is based on the different electrical properties of biological tissues [35,36].

We calculated the BMI by using body weight and body height (BMI=body weight [kg] / body height [m²]) and classified it according to Kromeyer-Hauschild et al [37]. This classification is based on German data and displays sex-specific BMI-for-age percentile curves from the age group of 15-79 years.

Due to the known weaknesses of the BMI, namely the missing discrimination of body fat and muscle mass [38], we calculated the height-normalized fat mass index (FMI=FM/m²) and fat-free mass index (FFMI=FFM/m²) based on the measured fat mass (FM), fat-free mass (FFM), and body height. The use of the FMI and FFMI instead of absolute values or percentages is recommended to avoid ambiguities when measuring FM and FFM themselves [39]. Consequently, the FMI (the higher, the more FM in relation to body height) and the FFMI (the higher, the more FFM in relation to body height) have been suggested to be better indicators when screening for diseases like obesity [39,40] or cardiometabolic risk factors [41] as well as physical activity [42] in adolescents compared to the BMI alone. Furthermore, a recent study showed that body composition parameters like FM are better predictors of body size dissatisfaction than the BMI [43].

We classified the FMI and the FFMI according to the MoMo (Motorik-Modul) study (Longitudinal Study of the German Health Interview and Examination Survey of Children and Adolescents; Studie von Kindern und Jugendlichen in Deutschland) [44], which published German sex-specific FMI-for-age and FFMI-for-age percentiles for the age group of 4-24 years. The study group recommends using the percentile curves of the normal weight sample as reference values for body composition assessment. Furthermore, they suggest using the 10th percentile as the cutoff value for low and the 90th percentile cutoff value for high FMI and FFMI. Consequently, the FMI and the FFMI between the 10th and 90th percentiles can be considered as normal.

### Statistical Analysis

Data were analyzed using SPSS (version 29.0.2.0; IBM Corp).

To illustrate descriptive parameters, the arithmetic mean and SD were stated for metric data. Interval variables or nominal-scaled variables were shown as frequencies. Due to the innovative nature of the approach and the exploratory design, no sample size calculations were performed and no a priori hypotheses were formulated.

The descriptive research included the descriptive analysis of the #fitspiration users and the following four questions versus corresponding body composition parameters (MoMo study normal weight [44]): (1) body image perception (body weight and body fat) versus FMI, (2) body-shaping behavior (weight reduction) versus FMI, (3) body image perception (muscularity) versus FFMI, and (4) body-shaping behavior (muscle building) versus FFMI.

### Ethical Considerations

The study protocol was submitted to the ethics committee of Vienna (MA 15-EK/21‐145-VK_NZ; June 16, 2021) and Lower Austria (email confirmation; October 15, 2021) and both stated that FIVE was not subject to mandatory submission.

FIVE was based on national and international ethical guidelines, above all the World Medical Association Declaration of Helsinki [45]. Participating adolescents received detailed written and oral information about the project as well as a comprehensive informed consent document. Furthermore, and in accordance with the recommendations of the Forum Ethics Commissions of Austria [46], we prepared separate informed consent forms for parents or legal guardians, who were informed comprehensively about the project.

The participants’ data were pseudonymized and assigned a fixed identification code. This code was stored safely, accessible only by the study coordinator and central staff members. The key for the coding was kept separately and inaccessible by the study coordinator. Data were stored and analyzed via the statistical software package SPSS at servers of the St. Pölten University of Applied Sciences, only used for this special study and not passed on to third parties. The handling of the data complied with the European General Data Protection Regulation, the Austrian Data Protection Act, and the recommendations of the Ethics Committee of Lower Austria.

Since the pupils participated in the project FIVE as part of their regular lessons, they received no compensation. Pupils who did not want to participate in the study were taught in classes that did not participate in the project FIVE. The teachers also participated in the project as part of their professional duties and, in accordance with Austrian law, were not allowed to receive additional payment. Nevertheless, and as a way of expressing gratitude, participating pupils received their BIA measurement results and were able to participate in workshops on the significance and interpretation of BIA measurements as well as gender and beauty standards on social media conducted by project staff members. The pupils had 2 options to choose from: either an online questionnaire only or a questionnaire plus anthropometric and BIA measurements.

## Results

### Participants

**Figure 1.**
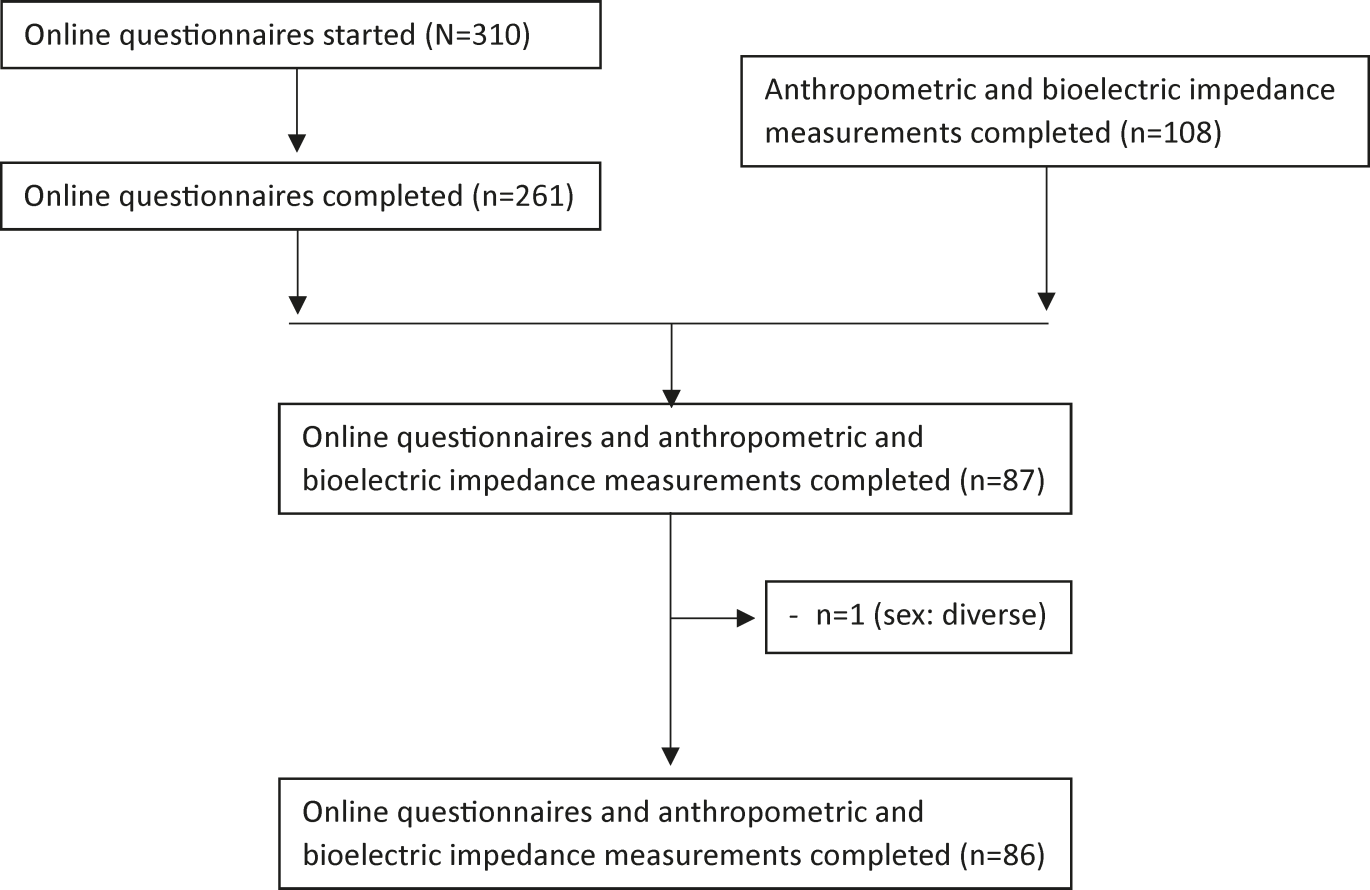
Participants’ flowchart.

Of the 6 participating upper secondary schools with about 1800 pupils, 310 pupils started with the online questionnaire (participation rate: approximately 20%). Among them, 261 pupils completed it (questionnaire completion rate: 84%) with an average completion time of 23.7 (SD 16) minutes. Overall, 108 out of 310 teenagers participated in the anthropometric and BIA measurements (anthropometric and BIA measurements participation rate is 35%). We combined data from the online questionnaire and the anthropometric and BIA measurements so that we had 87 complete datasets of questionnaire and anthropometric and BIA measurement. We excluded 1 person who identified as diverse because including 1 person in a separate gender category would not provide enough information. In addition, the used percentiles of FMI, FFMI [44], and BMI [37] are currently binary. Finally, we included 86 adolescents for further analysis (final inclusion rate turned out to be 28%). Figure 1 shows the participants’ flowchart.

In the next step, to examine the possible link between #fitspiration use and body image perception and body-shaping behaviors, we recoded the results from the questionnaire on #fitspiration use as follows: we defined participants as #fitspiration users if they selected the answer 4 (“I follow such content or influencers and will continue to do so”). #fitspiration nonusers, on the other hand, were those who ticked one of the first three answers (“I have never thought about following such content or influencers,” “I have thought about following such content or influencers. But I havent done it yet,” and “I thought about following such content or influencers. But I decided against it”). We classified those who selected the answer 5 (“I used to follow such content or influencers, but I stopped”) as former #fitspiration users.

In the last step, we summarized the 5 categories (2 negative, 1 neutral, and 2 positive) of the 2 questions regarding body image perception to 3 categories each (1 negative, 1 neutral, and 1 positive) because of distribution frequencies.

In total, 42/86 (49%) participants were female and 44/86 (51%) were male, aged 14-18 years (mean 16, SD 0.9 years). Apart from 1 participant, the sample lived in average-income (20/86, 23%) or well-off (65/86, 76%) families. The majority had no migration background (79/86, 92%). The 3 most used social media platforms, this means that social media were used multiple times per day, were WhatsApp (Meta Platforms, Inc; 67/86, 78%), Snapchat (Snap Inc.; 66/86, 77%), and Instagram (61/86, 71%).

With almost equal gender distribution, most of the participants did not use #fitspiration (59/86, 69%). Within the group of #fitspiration users (27/86, 31%), male participants were dominant (20/27, 74%). Former #fitspiration users represented the minority of the participants (8/86, 9%) and were mainly females (6/8, 75%).

Most participants were of normal weight (76/86, 88%; female: 37/42, 88%; male: 39/44, 89%). Furthermore, 6% (5/86; female: 2/42, 5%; male: 3/44, 7%) of participants were classified as overweight or obese, and 6% (5/86; female: 3/42, 7%; male: 2/44, 5%) of participants as severely underweight or underweight. The majority of participants had an FMI and an FFMI within the normal range (FMI: 51/86, 59%; female: 30/42, 71%; male: 21/44, 48%; FFMI: 50/86, 58%; female: 30/41, 71%; male: 20/44, 45%). Furthermore, 24/86 (28%; female: 4/42, 10%; male: 20/44, 46%) had a low FMI, and 29/86 (34%; female: 6/42, 14%; male: 23/44, 52%) had a high FMMI. Only 13% (11/86; female: 8/42, 19%; male: 3/44, 7%) had a high FMI, while 8% (7/86; female: 6/42, 14%; male: 1/44, 2%) had a low FFMI (see Table 1).

**Table 1.** Sample description inclusive of FMI and FFMI of the Motorik-Modul Study; sex-specific FMI and FFMI of normal-weight participants aged 14-17 years.

Variables	User (n=27)		Nonuser (n=51)		Former user (n=8)	
	Female (n=7)	Male (n=20)	Female (n=29)	Male (n=22)	Female (n=6)	Male (n=2)
Age (years), n (%)						
14	—^a^	1 (5)	3 (10)	1 (4)	—	—
15	2 (29)	2 (10)	11 (38)	5 (23)	—	—
16	1 (14)	10 (50)	11 (38)	8 (36)	3 (50)	—
17	4 (57)	7 (35)	3 (10)	5 (23)	3 (50)	1 (50)
18	—	—	1 (4)	3 (14)	—	1 (50)
Age (years), mean (SD)	16.3 (1)	16.2 (0.8)	15.6 (0.9)	16.2 (1.1)	16.5 (0.5)	17.5 (0.7)
FAS^b^, n (%)						
Low	—	—	1 (3)	—	—	—
Medium	1 (14)	3 (15)	10 (35)	6 (27)	—	—
High	6 (86)	17 (85)	18 (62)	16 (73)	6 (100)	2 (100)
Migration background, n (%)						
Yes	—	1 (5)	2 (7)	3 (14)	1 (17)	—
No	7 (100)	19 (95)	27 (93)	19 (86)	5 (83)	2 (100)
Social media use^c^, n (%)						
WhatsApp	7 (100)	16 (80)	21 (72)	15 (68)	6 (100)	2 (100)
YouTube	—	9 (45)	10 (35)	8 (36)	—	2 (100)
Instagram	7 (100)	17 (85)	19 (66)	14 (64)	4 (67)	—
Snapchat	7 (100)	15 (75)	23 (79)	14 (64)	6 (100)	1 (50)
TikTok	6 (86)	14 (70)	17 (59)	13 (59)	5 (83)	—
Pinterest	—	—	2 (7)	—	—	—
Facebook	1 (14)	—	—	—	—	—
others	1 (14)	4 (20)	2 (7)	6 (27)	1 (17)	1 (50)
BMI categorization, n (%)						
Severely underweight, underweight	—	—	3 (10)	2 (9)	—	—
Normal weight	7 (100)	18 (90)	24 (83)	19 (86)	6 (100)	2 (100)
Overweight, obesity	—	2 (10)	2 (7)	1 (5)	—	—
FMI^d^ categorization, n (%)						
<10th percentile	—	8 (40)	4 (14)	12 (55)	—	—
10th-90th percentile	4 (57)	11 (55)	20 (69)	8 (36)	6 (100)	2 (100)
>90th percentile	3 (43)	1 (5)	5 (17)	2 (9)	—	—
FMI (kg/m^2^), mean (SD)	6.3 (1.1)	2.8 (1.3)	5.1 (1.7)	3 (1.8)	5.7 (1.1)	5.2 (0.2)
FMI_MoMo_ (kg/m^2^), mean (SD)	5.1 (1.4)	3.9 (1.3)	5.1 (1.4)	3.9 (1.3)	5.1 (1.4)	3.9 (1.3)
FFMI^e^ categorization, n (%)						
<10th percentile	—	—	5 (17)	1 (5)	1 (17)	—
10th-90th percentile	7 (100)	8 (40)	19 (66)	11 (50)	4 (66)	1 (50)
>90th percentile	—	12 (60)	5 (17)	10 (45)	1 (17)	1 (50)
FFMI (kg/m^2^), mean (SD)	15.5 (0.8)	18.9 (1.4)	15.2 (1.2)	18.2 (1.6)	15.4 (1.4)	18.7 (1.9)
FFMI_MoMo_ (kg/m^2^), mean (SD)	15.6 (1.2)	16.9 (1.2)	15.6 (1.2)	16.9 (1.2)	15.6 (1.2)	16.9 (1.2)

a Not applicable.

b FAS: Family Affluence Scale (Low (score=0,1,2) indicates low affluence, medium (score=3,4,5) indicates middle affluence, and high (score=6,7,8,9) indicates high affluence).

c Social media use indicates multiple times per day; multiple answers possible.

d FMI: fat mass index.

e FFMI: fat-free mass index.

### Body Image Perception: Body Weight/Fat

Generally, 63% (54/86) of participants rated their body weight and body fat similar to the FMI. Participants with a low or average FMI (14/75, 19%) considered themselves too fat. Those with an average FMI (7/62, 11%) thought that they were too thin. In contrast to this, adolescents with a normal FMI had a better self-perception, and 65% (33/51) evaluated themselves as about normal weight.

Observed gender-related values are as follows: 75% (3/4) of female and 50% (10/20) of male teens with a low FMI overestimated their own body weight and body fat and thought that they were about normal weight or even too fat. Around 27% (8/30) of female participants and 14% (3/21) of male participants with a normal FMI considered themselves too fat. Overall, 88% (7/8) of female and 100% (3/3) of male participants with a high FMI rated themselves as too fat.

**Figure 2.**
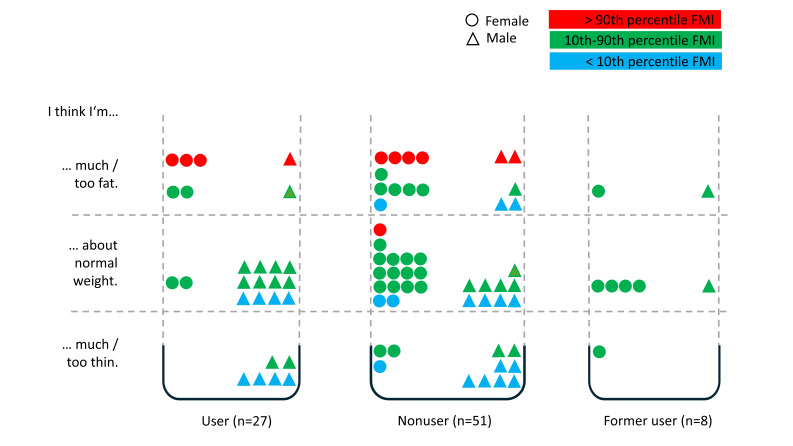
Body image perception (body weight and body fat) in relation to #fitspiration user status and FMI percentiles. FMI: fat mass index.

The association between the use of #fitspiration and body image perception appeared to be negligible (refer to Figure 2).

### Body-shaping Behavior: Weight Reduction

Generally, 43% (37/86) rated their body weight as fine (no related body-shaping behavior needed). Around 19% (16/86) of all participants implemented weight reduction behavior, and the same percentage thought that they should reduce body weight. Overall, 20% (17/86) of all participants thought that they should gain body weight.

Observed gender-related values are as follows: 24% (10/42) of female participants and 14% (6/44) of male participants implemented weight reduction behavior. Within the group of dieting participants (16/86, 19%), 40% (4/10) of females and 100% (6/6) of males were dieting although they had a low or normal FMI. In total, 5% (2/42) of female participants and 34% (15/44) of male participants were not on a diet because they thought that they should gain body weight. Altogether, 50% (21/42) of female participants and 36% (16/44) of male participants mentioned that they were not on a diet because their body weight was okay. Around 21% (9/42) of female participants and 16% (7/44) of male participants answered that while they were not on a diet, they should reduce body weight.

**Figure 3.**
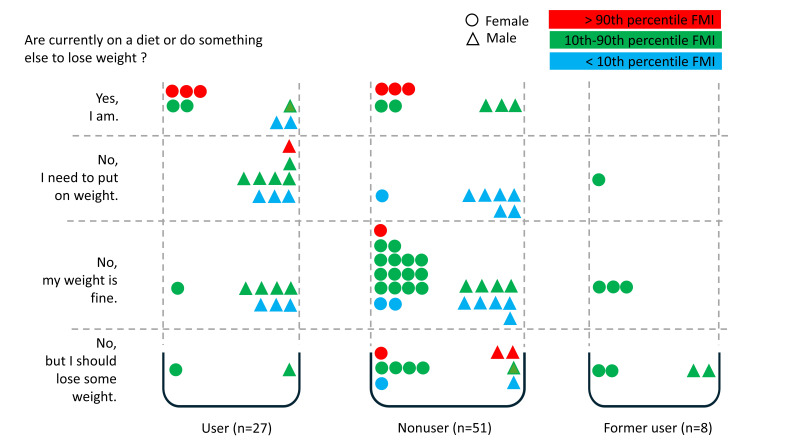
Body-shaping behavior (weight reduction) in relation to #fitspiration user status and FMI percentiles. FMI: fat mass index.

Regarding gender and #fitspiration use, 30% (8/27) of #fitspiration users—especially females (5/7, 71%; male: 3/20, 15%)—implemented weight reduction behavior. In comparison, no former #fitspiration user mentioned dieting, and 16% (8/51, female: 5/29, 17%; male: 3/22, 14%) of all nonusers were on a diet. #fitspiration nonusers and former users were more likely to state that they did not diet because they considered their body weight as fine (female user: 1/7, 14%; male user: 7/20, 35%; female nonuser: 17/29, 59%; male nonuser: 9/22, 41%; female former user: 3/6, 50%; male former user: 0/2, 0%; Figure 3).

### Body Image Perception: Muscularity

Generally, 31% (27/86) of all participants rated their muscularity according to their FFMI. A total of 55% (47/86) of all participants rated themselves as too unmuscular, although they had an FFMI within the normal range or above. All participants (86/86, 100%) with a low FFMI assessed themselves as too unmuscular (7/7).

Concerning observed gender-related values, 40% (17/42) of female participants versus 23% (10/46) of male participants evaluated their FFMI in accordance with the measurement results.

**Figure 4.**
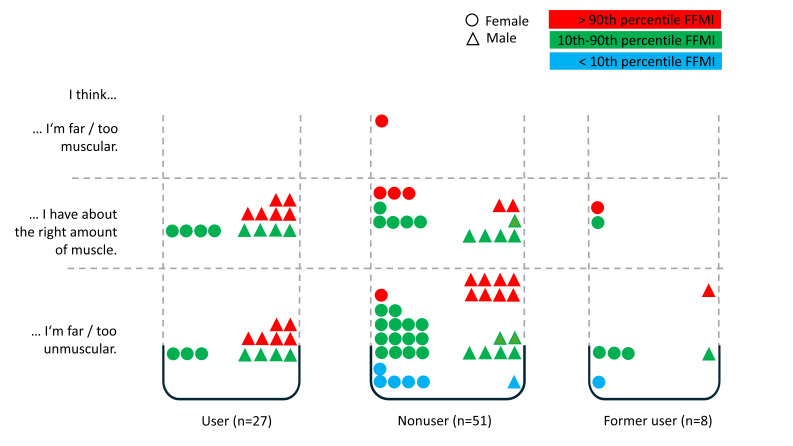
Body image perception (muscularity) in relation to #fitspiration user status and FFMI percentiles. FFMI: fat-free mass index.

Regarding gender and #fitspiration use, #fitspiration users (27/86, 31%) all had an FFMI within the normal range or above (female: 7/7, 100%; male: 20/20, 100%); but also, nonusers (female: 24/29, 83%; male: 21/22, 95%) and former users (female: 5/6, 83%; male: 2/2, 100%) showed similar values (refer to Figure 4).

### Body-shaping Behavior: Muscle Building

Generally, 69% (59/86) of all participants worked out or did something else to build muscle. Around 26% (22/86) of all participants thought that they should build muscle, 6% (5/86) of participants stated that their amount of muscle mass was okay, and no participant mentioned the wish to reduce muscle.

Observed gender-related values are as follows: Male participants were more likely to work out than female ones (female: 24/42, 57%; male: 35/44, 80%). On the other hand, female participants more often did not work out although they thought that they should build muscle (female: 15/42, 36%; male: 7/44, 16%).

**Figure 5.**
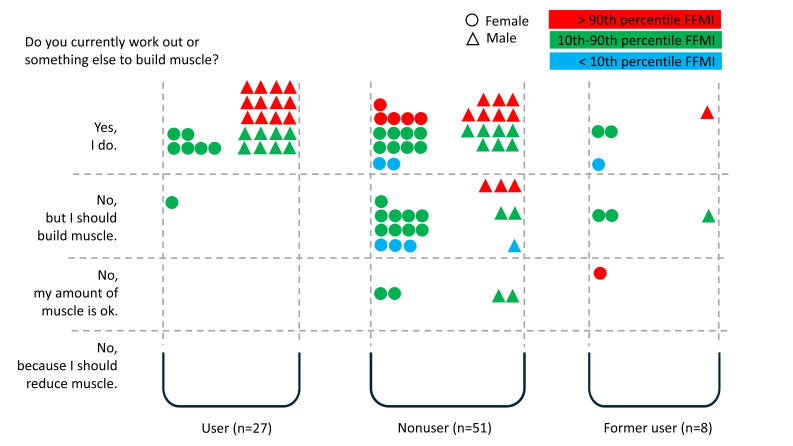
Body-shaping behavior (muscle building) in relation to #fitspiration user status and FFMI percentiles. FFMI: fat-free mass index.

Regarding gender and #fitspiration use, almost all #fitspiration users (26/27, 96%) worked out (female: 6/7, 86%; male: 20/20, 100%), and all users had an FFMI within the normal range or even higher. None of the #fitspiration users (0/27, 0%) answered that his or her amount of muscle mass was okay. We observed lower rates of those who worked out to build muscle within the other user groups, in particular nonusers (29/51, 57%; female: 15/29, 52%; male: 14/22, 64%) and former users (4/8, 50%; female: 3/6, 50%; male: 1/2, 50%). Overall, 35% (18/51; female: 12/29, 41%; male: 6/22, 27%) of nonusers and 38% (3/8; female: 2/6, 33%; male: 1/2, 50%) of former users thought that they should build muscle (see Figure 5).

## Discussion

### Main Findings and Comparison With Prior Work

The aim of this exploratory investigation study was to gain initial insights into gender-specific descriptions of adolescent #fitspiration user groups. In addition, we linked the results to body composition data, which allowed us to gain a deeper understanding of body image perceptions and body-shaping behaviors. Generally, muscularity seems to be of great importance for physical appearance. Male #fitspiration users showed greater dissatisfaction with muscularity, while female #fitspiration users were more concerned about their body weight. No #fitspiration user rated her or his amount of muscle mass as okay, although all of them had an average or high FFMI. Generally, body image perception was more accurate for FMI than for FFMI, suggesting some discrepancy in self-assessment of muscularity.

Considering the BMI, we observed a substantially lower number of adults with overweight or obesity in our sample than the Austrian HBSC average [47]. When compared to the normal-weight MoMo sample, our participants had a similar BMI. Furthermore, the FMI and FFMI of our participants were mainly within the normal range [44].

Almost two-thirds of the participants rated their body weight and body fat correctly compared to the FMI. This outcome is in line with nationwide cross-sectional studies where similar rates of adolescents rightly matched their BMI with their self-perceived weight [48,49]. Nevertheless, and similar to the Austrian HBSC study participants [50], approximately one-third of females and 1 out of 10 male participants with a low or normal FMI thought that they were too fat. This gender specificity is in line with a vast majority of studies, where female participants tend to overestimate their body weight [48,51-53]. Contrary to other studies, where adolescents with overweight or obesity underestimated their body weight [54,55], the majority of our participants above the 90th FMI percentile rated themselves correctly.

Concerning body-shaping behavior and its possible linkage to #fitspiration use, we observed the following: One in five of all adolescents, which is close to the Austrian HBSC average [34], stated that they were dieting or did something else to reduce body weight. In our investigation study, about one-third of #fitspiration users stated that they had implemented weight reduction behavior. In line with the results of body perception and body fat perception, female participants (whole sample: 10/42, 24%; #fitspiration users: 5/7, 71%) indicated weight reduction behavior more often than males (whole sample: 6/44, 14%; #fitspiration users: 3/20, 15%)—even if they were classified with a low or normal FMI. When compared with Austrian HBSC data, the percentage of young people who engaged in weight reduction behavior was similar [34]. This might raise the question of whether there is a connection between #fitspiration and body-shaping behavior. This has also been reported in other studies where the influence of #fitspiration had led to restrained eating behavior [56] and could increase eating disorder symptoms [26,57]. The majority of those who thought that they should gain weight were male participants. We assume that male participants had gaining muscle mass in mind rather than body weight. This is in line with literature that showed that boys are more likely to experience the pressure to increase muscle mass [58,59].

Content analysis of #fitspiration shows that these posts mainly show highly muscular men and women [60,61], which might lead to muscular-ideal internalization. Our findings might suggest that muscularity seemed to be of greater importance than thinness, and the participating adolescents seemed to find it more difficult to assess. Almost two-thirds of all participants rated themselves as too unmuscular or far too unmuscular, although they had an FFMI above the 10th percentile. This outcome seemed to be of greater significance to male participants. Most male participants with a high FFMI (12/23, 52%) belonged to the group of #fitspiration users, and nearly one-third of them (6/20) perceived themselves as too unmuscular. Besides self-perception, the importance of muscularity became apparent when it comes to strength training. Almost all participants said they worked out or thought that they should build muscle. Female participants were inactive more often, although they likewise thought that they should build muscle, whereas males worked out—or thought that they should—even though their FFMI was above the 90th percentile. Almost all #fitspiration users worked out, although they had an FFMI within the normal range or even higher. Although promoting exercise is highly recommended facing the enormous prevalence of overweight and obesity, the context of #fitspiration with its muscular ideals and training recommendations also raises the question of what motivates users to do strength training. Because appearance-based exercise motivation, which can be linked with #fitspiration [62], excessive or one-sided sports could lead to injuries, overload damage, and reduced immune function, besides psychological problems like eating disorders [63].

Our results indicate that girls have internalized the “new” beauty ideal, which includes thinness as well as muscularity [22,64]. The beauty ideal for boys still seemed to be “the more muscles, the better.” Although almost all participants seemed to aspire to these ideals, #fitspiration users appeared to be pursuing them even more. This is not surprising because the muscular ideals are core elements of #fitspiration [25,60] and could lead to the internalization of it [22]. But it is noteworthy that none of the 27 #fitspiration users stated that her or his amount of muscle is okay even though none of them had a low FFMI.

On the other hand, #fitspiration may help people move from thinking about doing sports to action. This assumption is derived from the observation that all male #fitspiration users and almost all female #fitspiration users were actively working out, while approximately one-third of the nonusers were just thinking about it. Similar to our findings, there are some studies indicating that #fitspiration might have positive influences on dietary change or physical activity [65,66].

### Limitations and Strengths

Overall, the main limitation regarding the results’ generalizability is the sampling—the study group (n=86) is relatively small and does not represent the general population, which is characteristic for exploratory investigation studies.

Moreover, there are further limitations concerning the sampling, which are, first, the majority of participants were of normal body weight, and our sample had lower rates of overweight or obesity than reported in the Austrian HBSC study [67]. This is of importance because the BMI is a known factor for influencing the accuracy of identifying a person’s weight status [68,69], body satisfaction and body dissatisfaction [68], or health behavior like dieting [70] or physical activity [71]–even in adolescents [54,55,72]. Therefore, it can be speculated that teen #fitspiration users with a more elevated BMI (originating from abnormal or excessive fat accumulation) may experience higher body dissatisfaction than the adolescents of this study. Furthermore, adolescents with overweight or obesity might implement fewer body-shaping behaviors, especially working out, due to the weight stigma than our mainly normal-weight sample. Second, most of the participants lived in well-off families without a migration background, which are known factors influencing body perception [73,74] and body weight control behavior [75]. Our results are not automatically transferable to adolescents of less affluent families or with migration backgrounds. Third, we did not quantify the intensity of #fitspiration use. Therefore, we cannot speculate concerning the amount of usage of #fitspiration and possible associations to body image perception and body-shaping behavior. Consequently, the results cannot be transferred to other adolescents using #fitspiration, which was not the objective of this study.

The strength of our study is first, that it adds additional perspectives regarding the use of #fitspiration and allows for the juxtaposition of the participants’ subjective assessment with objective measurements. Besides gender-specific descriptive analyses and differentiation between #fitspiration users and nonusers, we got first impressions of the perspective of former users. Second, anthropometric data were measured and not self-reported like at HBSC, which improves our data quality. Third, and to the best of our knowledge, our study is the first that conducted body composition measurements within different adolescent #fitspiration user groups. This allows us to gain first information on the potential relationship between #fitspiration use, body composition, self-perception of body shape, and body-shaping behaviors. But it does not allow a clear conclusion on whether exposure to this type of content leads to changes in behavior.

### Future Directions

Future studies should focus on the significance of muscularity for adolescent #fitspiration users and how they perceive their own muscle mass because it is questionable if #fitspiration use might foster body dissatisfaction and increase the risk of body dysmorphia. Is it possible that the enormous presence of supposedly perfect and more muscular bodies further increases the pressure on young people to conform to this ideal? How do extreme training, rigid eating habits, perhaps medically unsupervised supplementation, or even substance abuse affect the still-developing bodies of young people? Furthermore, research on #fitspiration should investigate how #fitspiration influences adolescents from diverse backgrounds, particularly those with migration backgrounds or lower socioeconomic status. Besides, it is the question of whether the characteristics of influencers, such as their professional qualification, type of sport, diet, or products they are presenting, affect #fitspiration users differently. Moreover, it is questionable whether a dose-response relationship regarding frequency and intensity of exposure and psychological or behavioral outcomes exists. Social media platform-specific dynamics should also be considered, as the effects of #fitspiration may vary across social media platforms due to differences in format, audience, and algorithmic exposure.

### Conclusion

In conclusion, health-related social media content #fitspiration is an important source of health information and body image perceptions for adolescents. This may influence health-related and body-shaping behaviors during this critical period of life. Our findings suggest that high muscle mass is an essential part of adolescents’ beauty ideals. Consequently, adolescents sought to achieve this through body-shaping behaviors—particularly strength training. #Fitspiration seemed to fuel these ideals and behaviors. Although the increasing prevalence of overweight and obesity makes exercise advisable, #fitspiration carries potential dangers for young people, especially body dissatisfaction, eating disorders, or physical damage caused by overtraining or incorrect training. This means that, on the one hand, more research is needed to understand the effects of #fitspiration on different target groups outside of laboratory settings. On the other hand, young people need to be equipped with the necessary knowledge and skills to use social media critically. To achieve this goal, all relevant stakeholders, especially families and schools, must collaborate. An increasingly rising amount of scientific effort, like the project FIVE, has been devoted to providing adolescents and stakeholders with age-appropriate, science-based courses [29], materials [76], and digital interventions [77,78].

In addition, health care professionals need to be aware that social media information and influencers are of great importance to adolescents and need to address this issue during their assessment process.

## Abbreviations

BIA: bioimpedance analysis
FAS: Family Affluence Scale
FFM: fat-free mass
FFMI: fat-free mass index
FIVE: #Fitspiration Image Verification
FM: fat mass
FMI: fat mass index
HBSC: Health Behaviour in School-aged Children
MoMo: Motorik-Modul Longitudinal Study of the KiGGS study
UNECE: United Nations Economic Commission for Europe
